# Circ_0029803 serves as the sponge of miR-216b-5p to promote the progression of colorectal cancer by regulating SKIL expression

**DOI:** 10.1186/s12957-021-02368-2

**Published:** 2021-09-03

**Authors:** Linfei Huang, Lei Zhu, Sheng Pan, Jing Xu, Miao Xie, Wei Wang, Ganlin Xia

**Affiliations:** Department of Gastrointestinal Surgery, Wuhan Puren Hospital Affiliated to Wuhan University of Science and Technology, No.1 Benxi Street, Qingshan District, Wuhan City, 430080 Hubei Province People’s Republic of China

**Keywords:** Colorectal cancer, circ_0029803, miR-216b-5p, SKIL

## Abstract

**Background:**

Circular RNA 0029803 (circ_0029803) was found to be upregulated in colorectal cancer (CRC) tissues, but its function and underlying molecular mechanism are not studied in CRC.

**Methods:**

The expression levels of circ_0029803, microRNA-216b-5p (miR-216b-5p), and ski-oncogene-like (SKIL) were measured by quantitative real-time polymerase chain reaction (qRT-PCR). RNase R treatment was used to affirm the existence of circ_0029803. Cell proliferation, apoptosis, migration, and invasion were assessed by colony formation, flow cytometry, and Transwell assays, respectively. A glucose and lactate assay kit was used to detect glucose consumption and lactate production. Western blot was applied to analyze the levels of all proteins. Dual-luciferase reporter assay was performed to assess the relationship between miR-216b-5p and circ_0029803 or SKIL. Tumor xenograft models were established to elucidate the effect of circ_0029803 in vivo.

**Results:**

Circ_0029803 expression was enhanced in CRC tissues and cells, and the 5-year overall survival rate of patients with high circ_0029803 expression was substantially reduced. Circ_0029803 depletion retarded proliferation, migration, invasion, EMT and glycolysis of CRC cells in vitro as well as the tumor growth in vivo. Mechanically, circ_0029803 could serve as miR-216b-5p sponge to regulate its expression, and miR-216b-5p knockdown reversed the inhibition of si-circ_0029803 on the malignant behaviors of CRC cells. Additionally, as the target mRNA of miR-216b-5p, SKIL could counteract the inhibitory effect of miR-216b-5p on the development of CRC cells. Importantly, silencing circ_0029803 reduced SKIL expression via sponging miR-216b-5p.

**Conclusion:**

Circ_0029803 knockdown hindered proliferation, migration, invasion, EMT, and glycolysis and promoted apoptosis in CRC cells by modulating the miR-216b-5p/SKIL axis.

## Introduction

Colorectal cancer (CRC) is a type of malignant tumor, and its incidence is on the rise in both men and women because of irregular lifestyle and dietary habits [1]. Despite significant advances in clinical treatment, patients with CRC have a poor prognosis due to advanced diagnosis and rapid metastasis of the tumors [2]. Therefore, it is urgently necessary to understand the molecular mechanisms that induce CRC growth and metastasis.

Circular RNA (circRNA) is a special type of non-coding RNA (ncRNA), which is stable and abundant due to its covalently closed structure [3], and it has been widely introduced to be involved in the occurrence and development of cancers [4, 5]. Lately, circRNAs were found to be differentially expressed in CRC tissues compared to adjacent normal tissues and associated with the biological behaviors of cancer cells [6–8]. Zhang et al. suggested that circRNA dedicator of cytokinesis 1 (circ_DOCK1) acted miR-132-3p sponge to regulate the expression of ubiquitin-specific protease 11 (USP11), thereby regulating cell growth, metastasis, and apoptosis in CRC [9]. Besides, hsa_circ_0000231 promoted cell glycolysis and progression in CRC by regulating miR-502-5p/Myosin VI (MYO6) axis [10]. Circ_0082182 was reported to exert its tumor promoter function in CRC cells by sponging miR-411 or miR-1205 to activate the Wnt/β-catenin pathway [11]. Furthermore, circ_0001136/miR-1205/glutamate ionotropic receptor kainate type subunit 3 (GRIK3) regulated the malignant progression of CRC in vitro and in vivo [12]. In addition, overexpression of circLgr4 facilitated self-renewal of CRC stem cells, tumorigenesis, and invasion [13]. Although circRNAs related to CRC progression have been widely reported, there are still numerous circRNAs whose functions have not been explored. For example, Li et al. found that circ_0029803 was upregulated in CRC tissues through RNA-Seq [14]. However, its functions and potential mechanisms have never been studied.

CircRNA contains microRNA (miRNA) response elements (MREs), which can act as a competitive endogenous RNA (ceRNA) of miRNA to regulate the expression of target mRNAs [15]. Dysregulation of miRNA expression has also been found to be closely related to the development of CRC. For instance, miR-708 [16] and miR-193a-3p [17] could act as tumor suppressors to inhibit the progression of CRC, while miR-501-3p [18] and miR-17 [19] could promote the development of CRC. MiR-216b-5p was reported to act as a tumor-suppressive RNA to suppress tumorigenesis in pancreatic cancer [20] and breast cancer [21]. Furthermore, Sun et al. revealed that miR-216b-5p might be related to CRC cell activity and glycolysis, but the specific mechanisms of miR-216b-5p in CRC remains unclear. Ski-oncogene-like (SKIL or SnoN), an inverse regulator of TGF-β signaling, has been shown to function as an oncogene in oral cancer [22]. However, it served as a cancer suppressor in the early stages of some human cancers [23], which aroused our concern to investigate the role of SKIL in CRC.

In the current study, the expression of circ_0029803 in CRC tissues and cells was detected, and we explored its biological functions in vitro and in vivo. Moreover, a novel molecular mechanism mediated by circ_0029803 in CRC was investigated to further understand the pathogenesis of CRC.

## Materials and methods

### Tissue samples and cell culture

Tumor tissues and paracancerous tissues from 62 patients with CRC were collected at Wuhan Puren Hospital Affiliated to Wuhan University of Science and Technology. The collection of tissue samples was permitted by the Human Ethics Committee of Wuhan Puren Hospital Affiliated to Wuhan University of Science and Technology, and these CRC patients provided the written informed consents. The collected CRC tissues were divided into two groups based on the expression of circ_0029803: one group with high circ_0029803 expression (*n*=35) and another group with low circ_0029803 expression (n=27).

CRC cell lines, HCT-116 and SW480, were bought from Procell (Wuhan, China), and human colon epithelial cell line, NCM460, was obtained from SUER (Shanghai, China). All cell lines were hatched in Roswell Park Memorial Institute 1640 (RPMI 1640, Hyclone, South Logan, UT, USA) medium containing 10% fetal bovine serum (FBS, Hyclone) at 37°C in an incubator containing 5% CO_2_.

### Quantitative real-time polymerase chain reaction (qRT-PCR)

Trizol reagent (Invitrogen, Carlsbad, CA, USA) was applied for total RNA extraction from CRC cell lines and tissues. After quality detection, the complementary DNA (cDNA) was synthesized using PrimeScript RT kit (Takara, Dalian, China). Subsequently, qRT-PCR was carried out using PrimeScript RT Master Mix (Takara). Glyceraldehyde-3-phosphate dehydrogenase (GAPDH) or U6 was used as internal reference of circ_0029803 and SKIL or miR-216b-5p, and the relative expression level was analyzed by the 2^−∆∆Ct^ method. The primer sequences used in this paper were as follows: circ_0029803, Forward (F): 5′-AGTTACCTCGGGGAATGGTGA-3′, Reverse (R): 5′-AATGCTATTTCTCTACATGCCGAC-3′. GAPDH, F: 5′-GGAGTCCACTGGCGTCTTCA-3′, R: 5′-GGTTCACACCCATGACGAAC-3′. miR-216b-5p, F: 5′-GGGGAAATCTCTGCAGGCAA-3′, R: 5′-CAGTGCAGGGTCCGAGGT-3′. U6, F: 5′-CTCGCTTCGGCAGCACA-3′, R: 5′-AACGCTTCACGAATTTGCGT-3′. SKIL, F: 5′-AGAGGCTGAATATGCAGGACA-3′, R: 5′-CCAAAGCAAGCAACAAACAA-3′. These primer sequences were synthesized by GenePharma (Shanghai, China).

### Nuclear-cytoplasmic fractionation and RNase R treatment

The RNA of cytoplasm and nuclear was separated by PARIS kit (Invitrogen). Briefly, HCT-116 and SW480 cells were collected and centrifuged, the supernatant was used to extract the RNA of cytoplasm, and the precipitation was applied to acquire nuclear RNA. Besides, GAPDH and U6 were used as the controls of cytoplasm and nuclear, respectively.

RNA was isolated from HCT-116 and SW480 cells, and it was treated with or without RNase R (Epicentre Technologies, Madison, WI, USA) at 37°C for 30 min. qRT-PCR was then used to examine the stability of circ_0029803.

### Transfection

For circ_0029803 interference, small interfering RNA targeting circ_0029803 (si-circ_0029803) and the control si-NC were synthesized by GenePharma. Moreover, miR-216b-5p mimics (miR-216b-5p), miR-216b-5p inhibitor (anti-miR-216b-5p), and their matched controls (miR-NC and anti-miR-NC) were designed by GenePharma. The overexpression plasmid of SKIL and its negative control (vector), lentivirus-sh-circ_0029803 and control sh-NC were provided by Fenghui Biotechnology Company (Changsha, China). Cell transfection was conducted using Lipofectamine 3000 (Invitrogen).

### Colony formation assay

The transfected HCT-116 and SW480 cells were evenly spread in 6-well plates (200 cells/well) and placed in a 37°C incubator. 2 weeks later, the cells were fixed with methanol and stained with 0.5% crystal violet (Beyotime, Beijing, China) for 20 min, and the number of colonies was then counted using a light microscope.

### Cell apoptosis assay

The apoptosis of HCT-116 and SW480 cells was detected using the Annexin V fluorescein isothiocynate (FITC)/propidium iodide (PI) apoptosis detection kit (Beyotime). The 6-well plates with transfected CRC cells were placed in a 37°C incubator for 48 h. Next, cells were harvested, FITC and PI were applied to stain the cells for 20 min in the absence of light, and the cells were analyzed by flow cytometry (FlowCam, Shanghai, China).

### Analysis of cell migration and invasion

Transwell assay was used to analyze the migration and invasion ability of cells. The difference between them was that the Transwell chamber was coated with Matrigel (Solarbio, Beijing, China) during the invasion experiment. The transfected CRC cells in 200 μL of serum-free medium were seeded into the upper chamber, and 500 μL of complete medium was added into the bottom chamber. Cells were then incubated at 37°C for 24 h. Cells migrated or invaded through the chamber were stained with 0.1% crystal violet (Beyotime) for 20 min and counted under an inverted microscope.

### Western blot assay

CRC tissues and transfected CRC cells were lysed using RIPA (Beyotime). The extracted protein was isolated and transferred to the polyvinylidene difluoride (PVDF) membranes (Beyotime). After blocking with skim milk powder for 2 h, the membranes were incubated overnight with primary antibodies at 4°C. After washing with TBST, horseradish peroxidase (HRP)-labeled secondary antibody (1:5000, Abcam, Cambridge, MA, USA) was applied to incubate the membranes. Finally, the bands were visualized using an ECL detection kit (Beyotime). The primary antibodies were used as follows: E-cadherin (1:200, Abcam), N-cadherin (1:500, Abcam), Vimentin (1:200, Abcam), Hexokinase2 (HK2, 1:1000, Abcam), SKIL (0.5 μg/ML, Abcam), and β-actin (1:3000, Abcam).

### Glucose consumption and lactate production measurement

The transfected HCT-116 and SW480 cells were tiled into 6-well plates and incubated for 48 h. Subsequently, the concentrations of glucose and lactate in the supernatant of medium were examined by a glucose and lactate assay kit (Sigma-Aldrich, St. Louis, MO, USA). Glucose consumption was calculated by subtracting the concentration of glucose in the medium at a specific time point from the concentration of glucose in the fresh complete medium. Lactate production was obtained by subtracting the concentration of lactate in the fresh original medium from the concentration of lactate in the collected supernatant.

### Dual-luciferase reporter assay

For circ_0029803, the sequences of wild-type (WT) or mutant (MUT) containing miR-216b-5p binding sites or not were inserted into the pmirGLO vector (Promega, Madison, WI, USA). For SKIL, the 3′ untranslated region (3′UTR) of WT or MUT containing miR-216b-5p binding sites or not were inserted into the pmirGLO vector. Then, these report plasmids were respectively co-transfected with miR-216b-5p or miR-NC into CRC cell lines. At 36 h after the transfection, the luciferase activity was checked by a dual-luciferase reporter kit (Promega).

### Tumor xenografts

The 4-week-old BALB/c nude mice were used to construct a tumor xenograft model. SW480 cells were first stably transfected with sh-circ_0029803 or control sh-NC, and then, the transfected SW480 cells were inoculated into the nude mice, classified into sh-circ_0029803 group and sh-NC group. Tumor volume was measured and calculated every week. Five weeks later, all mice were euthanized and the tumor tissues were weighed and collected for the detection of circ_0029803, miR-216b-5p and SKIL expression levels. The experiment was permitted by the Animal Research Committee of the Wuhan Puren Hospital Affiliated to Wuhan University of Science and Technology.

### IHC staining analysis

Five-micrometer-thickness tissue sections were prepared, followed by dewaxing and blocking. Then, tissue sections were probed with the primary antibody targeting SKIL at 4°C overnight and proved with the secondary antibody for 1 h. Subsequently, tissue sections were stained using a 3, 3′-diaminobenzidine (DAB) kit (Abcam).

### Statistical analysis

All data were processed by using GraphPad Prism 7 software (GraphPad Inc., La Jolla, CA, USA). All data were repeated at least three times and appeared as the mean ± standard deviation (SD).The difference between two groups was analyzed by Student’s *t*-test, and multiple group comparisons were assessed using one-way analysis of variance (ANOVA) analysis. *P* < 0.05 was regarded as a statistically distinct difference.

## Results

### Increased circ_0029803 expression was associated with the progression of CRC

**Fig. 1:**
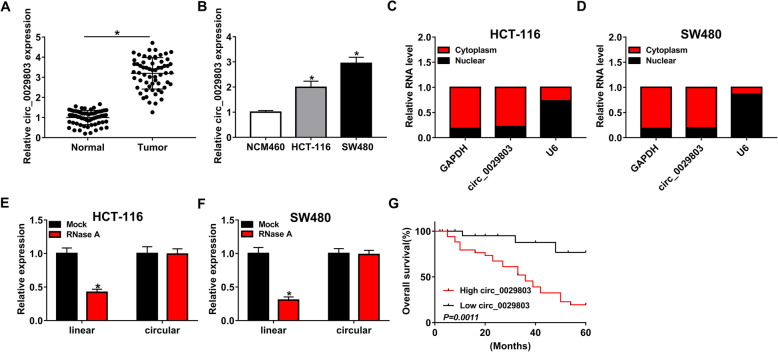
Increased circ_0029803 expression was associated with the progression of CRC. **A** Circ_0029803 expression in CRC tissues and normal tissues was detected by qRT-PCR. **B** Circ_0029803 expression in CRC cell lines (HCT-116 and SW480) and NCM460 cell line was measured by qRT-PCR. **C**, **D** The level of circ_0029803 in cytoplasm and nuclear of HCT-116 and SW480 cells was checked by qRT-PCR. **E**, **F** QRT-PCR was used to examine the abundance of circ_0029803 and linear mRNA in CT-116 and SW480 cells treated with RNase R. **G** Kaplan-Meier survival curve was used to analyze the 5-year overall survival of CRC patients with high circ_0029803 expression or low circ_0029803 expression. *P<0.05

To explore the role of circ_0029803 in CRC, its expression in CRC tissues and cell lines was measured by qRT-PCR. The results showed that circ_0029803 expression was aggrandized in CRC tissues (*n* = 62) compared with adjacent normal tissues (Fig. 1A). As displayed in Table 1, we analyzed the correlation between circ_0029803 expression and clinicopathological features of the 62 patients. The data showed that high expression of circ_0029803 was related to larger tumor sizes (*P*=0.011). Consistently, the expression of circ_0029803 was increased in CRC cell lines (HCT-116 and SW480) compared to normal colon epithelial cell line NCM460 (Fig. 1B). The cell nuclear-cytoplasmic fraction assay showed that circ_0029803 was primarily located in the cytoplasm of HCT-116 and SW480 cells (Fig. 1C, D). RNase R, an exoribonuclease, has been widely introduced to degrade linear RNA from its 3′ to 5′ end, but does not act on circRNA [24]. As shown in Fig. 1E, F, circ_0029803 was resistant to RNase R digestion, while RNase R could degrade linear mRNA. Besides, The Kaplan-Meier overall survival (OS) curve showed that the 5-year survival rate of patients with high circ_0029803 expression was lower than that of patients with low circ_0029803 expression (Fig. 1G). The data suggested that circ_0029803 might be a key factor in the regulation of CRC progression.

**Table 1:** Correlations between circ_0029803 expression in plasmas and clinical characteristics in patients with CRC

Parameter	Case	circ_0029803 expression		*P* value^a^
		Low (*n*=27)	High (*n*=35)	
Age (years)				0.116
≤60	32	17	15	
>60	30	10	20	
Sex				0.0625
Female	33	18	15	
Male	29	9	20	
Histological grade				0.147
High	28	15	13	
Middle-low	34	12	22	
Tumor size				<0.0001*
≤5 cm	33	24	9	
>5 cm	29	3	26	
TNM stages				0.014*
I–II	35	20	15	
III–IV	27	7	20	
Lymph node metastasis				<0.0001*
Negative	29	21	8	
Positive	33	6	27	

*TNM*, tumor-node-metas-tasis; *P < 0.05; ^a^Chi-square test

### Circ_0029803 knockdown suppressed the progression of CRC cells in vitro

**Fig. 2:**
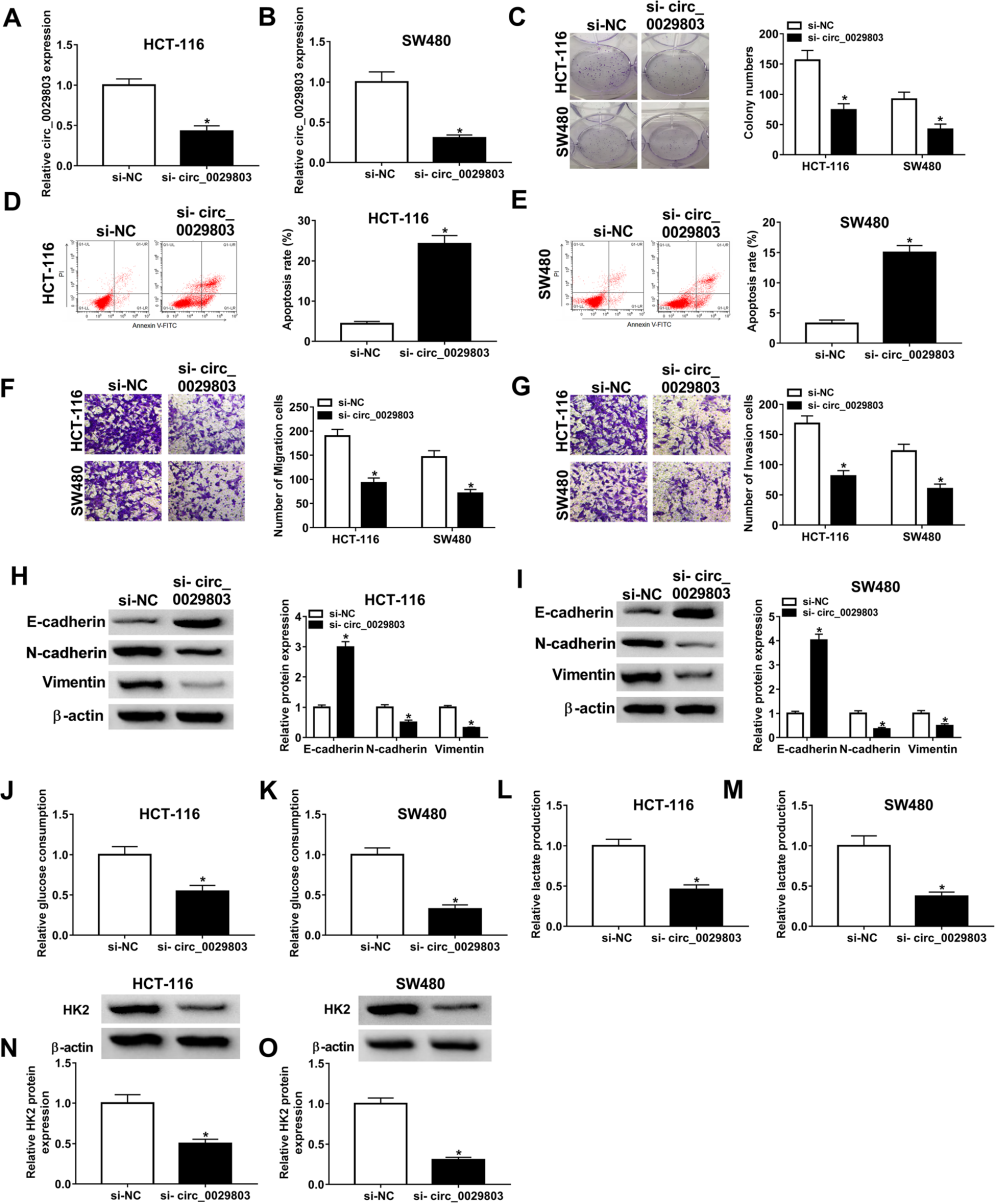
Circ_0029803 knockdown suppressed the proliferation, migration, invasion, EMT, and glycolysis of CRC cells in vitro. After HCT-116 and SW480 cells were transfected with si-NC or si-circ_0029803. **A**, **B** Circ_0029803 expression was measured by qRT-PCR. **C** The cells proliferation was assessed by colony formation assay. **D**, **E** The cells apoptosis rate was determined by flow cytometry. **F**, **G** The migration and invasion of cells were detected by Transwell assay. **H**, **I** The protein levels of E-cadherin, N-cadherin, and Vimentin were checked by western blot. **J**–**M** Glucose consumption and lactate production of cells were measured by a glucose and lactate assay kit. **N**, **O** The protein level of HK was checked by western blot. *P<0.05

Due to the high expression of circ_0029803 in CRC, we knocked down circ_0029803 in CRC cells to explore its function. As displayed in Fig. 2A, B, circ_0029803 expression was evidently decreased after the transfection of si-circ_0029803 into HCT-116 and SW480 cells. Then, colony formation assay demonstrated that the cloning ability of HCT-116 and SW480 cells was inhibited after the downregulation of circ_0029803 (Fig. 2C). The results of flow cytometry assay showed that circ_0029803 knockdown augmented the apoptosis rate of the cells (Fig. 2D, E). Transwell assay further indicated that when circ_0029803 expression was silenced in HCT-116 and SW480 cells, cell migration and invasion abilities were reduced (Fig. 2F, G). It is well known that EMT plays a crucial role in the process of tumor metastasis [25]. Therefore, we also studied the effect of si-circ_0029803 on the expression of EMT marker proteins, including E-cadherin, N-cadherin, and Vimentin, by western blot in HCT-116 and SW480 cells. As shown in Fig. 2H, I, interference with circ_0029803 strikingly increased the protein expression of E-cadherin and decreased the protein levels of N-cadherin and Vimentin, suggesting that circ_0029803 knockdown restrained EMT of CRC cells. In addition, the knockdown of circ_0029803 significantly reduced glucose consumption (Fig. 2J, K), lactate production (Fig. 2L, M), and the protein level of HK2 (Fig. 2N, O) in HCT-116 and SW480 cells. These results indicated that downregulation of circ_0029803 repressed colony formation, migration, invasion, EMT, and glycolysis of CRC cells, and induced cell apoptosis in vitro.

### Circ_0029803 served as a sponge for miR-216b-5p

**Fig. 3:**
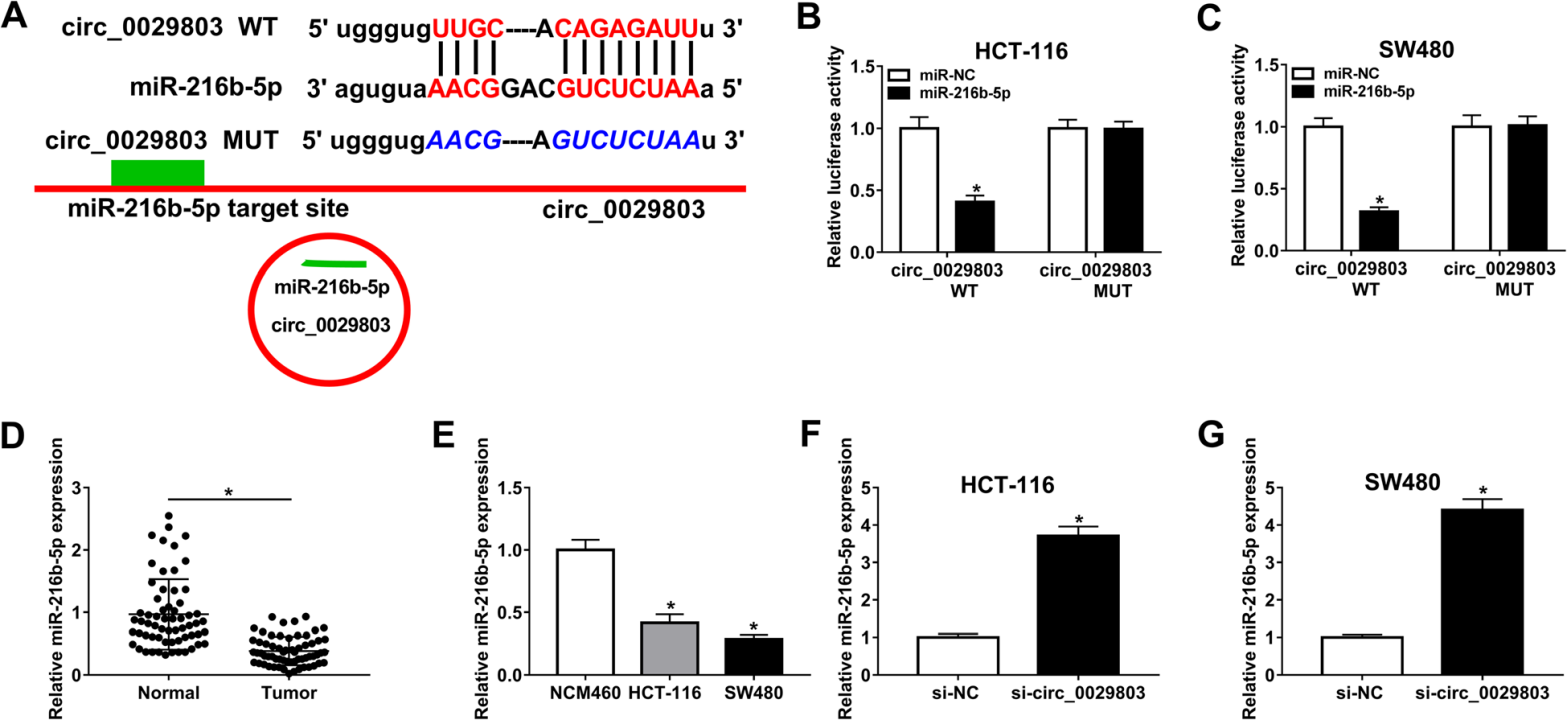
Circ_0029803 served as a sponge for miR-216b-5p. **A** The binding sites between circ_0029803 and miR-216b-5p were predicted by StarBase v2.0 and the mutant sequences of circ_0029803 were shown. **B**, **C** The luciferase activity was analyzed in HCT-116 and SW480 cells co-transfected with circ_0029803 WT or circ_0029803 MUT and miR-216b-5p or miR-NC by dual-luciferase reporter assay. **D**, **E** MiR-216b-5p expression in CRC tissues and cells compared to that in normal tissues and cells was detected by qRT-PCR. **F**, **G** MiR-216b-5p expression in HCT-116 and SW480 cells transfected with si-NC or si-circ_0029803 was checked by qRT-PCR. *P<0.05

CircRNAs have been reported to exert their biological functions as miRNA sponges to regulate miRNA expression [26]. To investigate whether circ_0029803 could act as a sponge for miRNAs in CRC cells, we predicted the possible binding miRNAs of circ_0029803 by StarBase v2.0, and miR-216b-5p was found to have complementary sites that bind to circ_0029803 (Fig. 3A). To validate the direct binding between them, dual-luciferase reporter assay was performed in HCT-116 and SW480 cells. The results revealed that miR-216b-5p distinctly decreased the luciferase activity in cells transfected with circ_0029803 WT compared to control, whereas did not show significant effect on that of circ_0029803 MUT (Fig. 3B, C). Next, we examined the expression of miR-216b-5p in CRC tissues and cells by qRT-PCR. The data showed that miR-216b-5p expression was downregulated in CRC tissues and cells compared with normal tissues and cells (Fig. 3D, E). Moreover, circ_0029803 knockdown increased miR-216b-5p expression in HCT-116 and SW480 cells (Fig. 3F, G). The above results implied that circ_0029803 directly targeted miR-216b-5p and regulated its expression in CRC cells.

### Circ_0029803 knockdown suppressed the progression of CRC cells by targeting miR-216b-5p

**Fig. 4:**
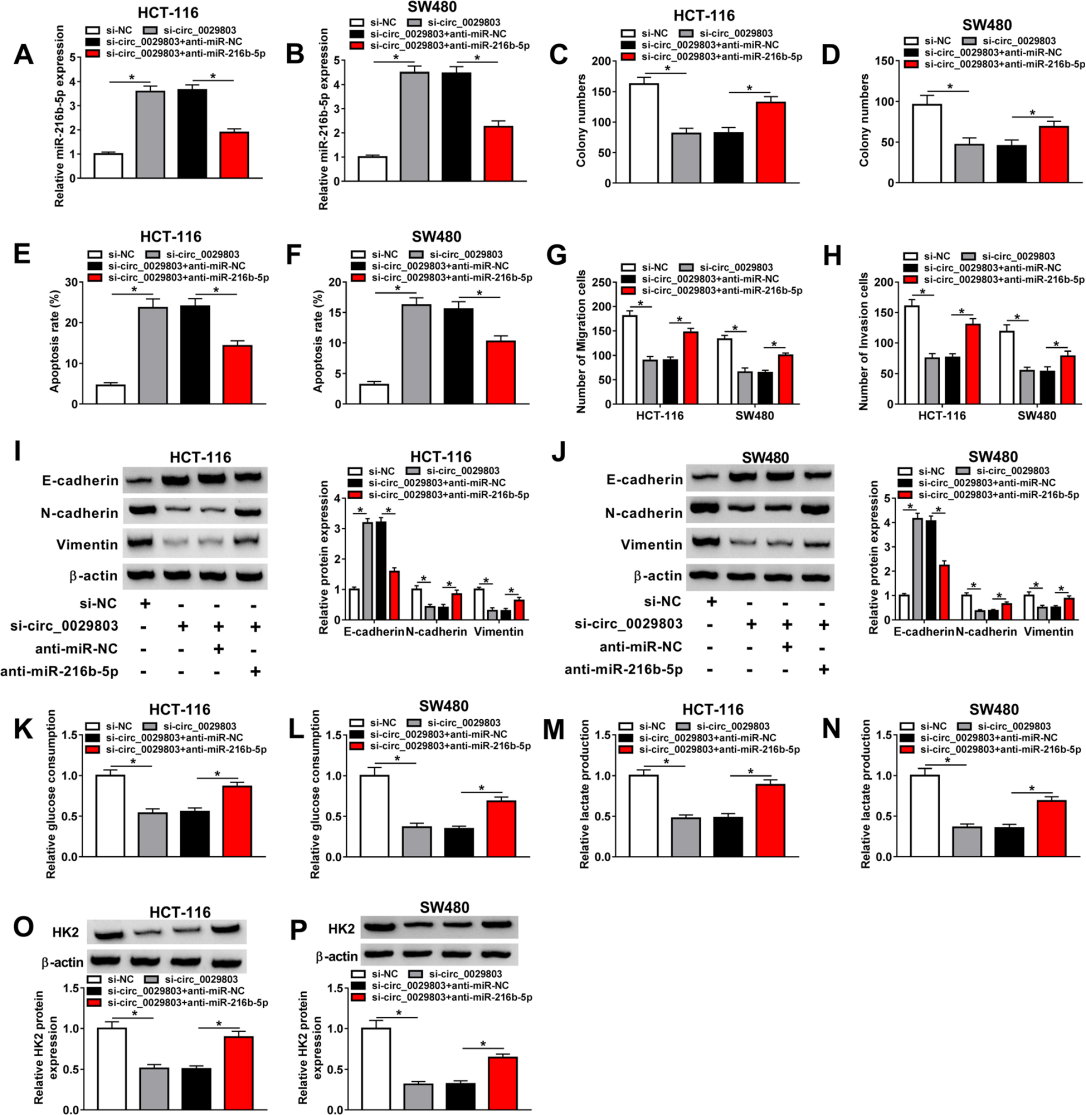
Circ_0029803 knockdown suppressed the progression of CRC cells by targeting miR-216b-5p. **A**, **B** MiR-216b-5p expression in HCT-116 and SW480 cells transfected with si-NC, si-circ_0029803, si-circ_0029803 + anti-miR-NC, or si-circ_0029803 + anti-miR-216b-5p was examined by qRT-PCR. **C**, **D** The proliferation of transfected HCT-116 and SW480 cells was measured using colony formation assay. **E**, **F** The apoptosis rate of transfected HCT-116 and SW480 cells was detected by Flow cytometry. **G**, **H** The migration and invasion of transfected HCT-116 and SW480 cells were detected by Transwell assay. **I**, **J** The levels of E-cadherin, N-cadherin, and Vimentin were checked by western blot. **K**–**N** Glucose consumption and lactate production of transfected HCT-116 and SW480 cells were measured using a glucose and lactate assay kit. **O**, **P** The protein level of HK in transfected HCT-116 and SW480 cells was measured by western blot. *P<0.05

We have proved that miR-216b-5p was a target miRNA of circ_0029803. Next, we investigated whether miR-216b-5p could affect the function of circ_0029803 in CRC cells by the recovery experiments. First, qRT-PCR results showed that anti-miR-216b-5p could reverse the upregulation effect of si-circ_0029803 on miR-216b-5p expression in HCT-116 and SW480 cells (Fig. 4A, B). Colony formation, flow cytometry, and Transwell assays were respectively demonstrated that interference with miR-216b-5p in HCT-116 and SW480 cells alleviated the inhibition of circ_0029803 knockdown on cell clone formation (Fig. 4C, D), migration, and invasion (Fig. 4G, H), and partially eliminated the promotion effect of circ_0029803 knockdown on cell apoptosis (Fig. 4E, F). Furthermore, the effect of si-circ_0029803 on levels of EMT marker proteins described in Fig. 2H, I could also be overturned by anti-miR-216b-5p (Fig. 4I, J). Simultaneously, the inhibitory effect of si-circ_0029803 on glucose consumption (Fig. 4K, L), lactate production (Fig. 4M, N), and the protein level of HK2 (Fig. 4O, P) in HCT-116 and SW480 cells was counteracted by silencing miR-216b-5p. The results supported that circ_0029803 modulated the progression of CRC cells by acting as a sponge of miR-216b-5p.

### Circ_0029803 positively modulated SKIL expression via sponging miR-216b-5p

**Fig. 5:**
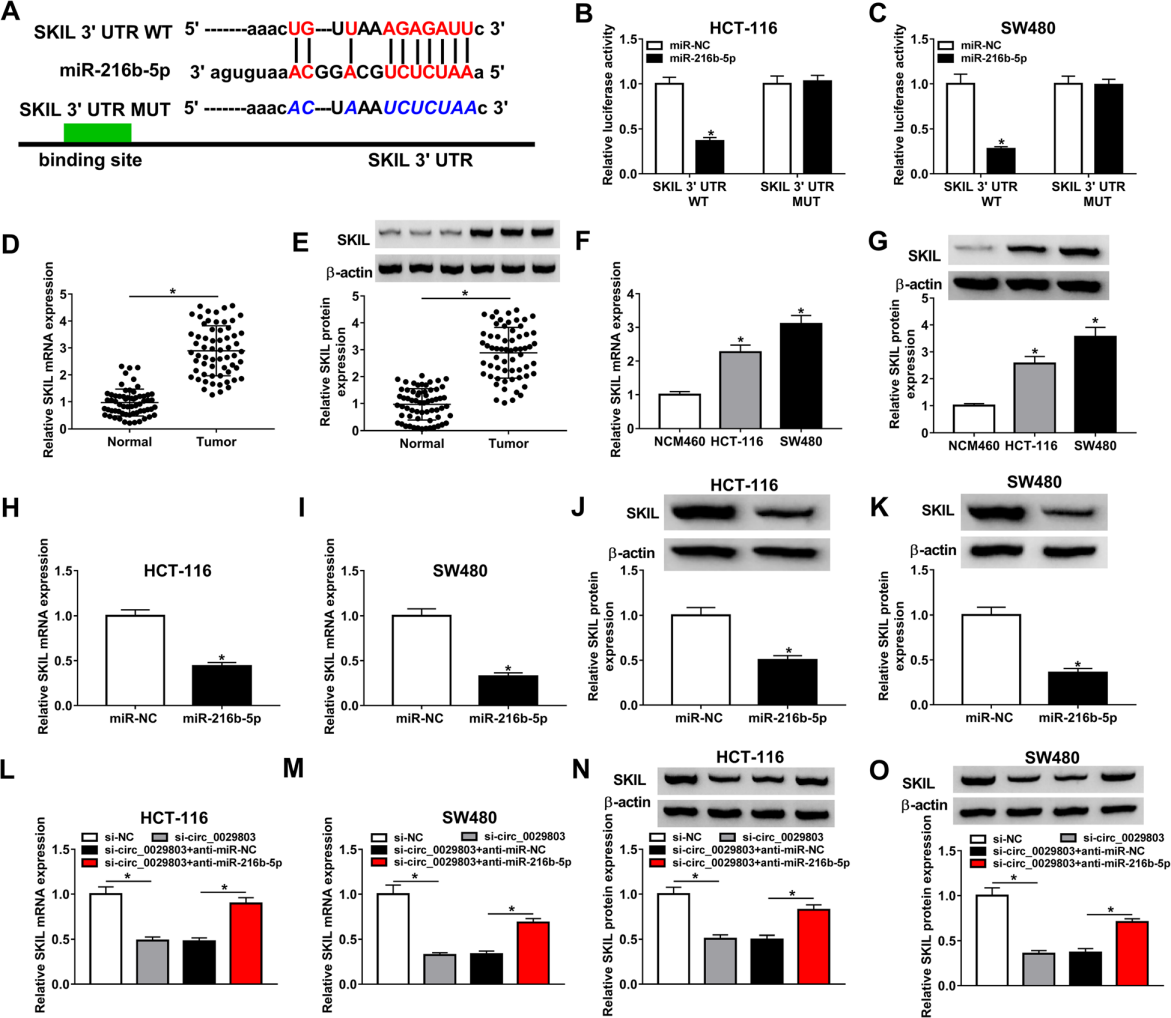
Circ_0029803 positively modulated SKIL expression via sponging miR-216b-5p. **A** The binding sites between miR-216b-5p and SKIL were predicted by StarBase v2.0. **B**, **C** The luciferase activity was assessed in HCT-116 and SW480 cells co-transfected with SKIL 3′ UTR WT or SKIL 3′ UTR MUT and miR-216b-5p or miR-NC by dual-luciferase reporter assay. **D**–**G** SKIL mRNA and protein levels in CRC tissues and cells compared with normal tissues and cells were detected by qRT-PCR and western blot, respectively. **H**–**K** The mRNA and protein levels of SKIL in HCT-116 and SW480 cells transfected with miR-NC or miR-216b-5p were examined by qRT-PCR and western blot, respectively. **L**–**O** The mRNA and protein levels of SKIL in HCT-116 and SW480 cells transfected with si-NC, si-circ_0029803, si-circ_0029803 + anti-miR-NC, or si-circ_0029803 + anti-miR-216b-5p were examined by qRT-PCR and western blot, respectively. *P<0.05

Subsequently, we searched the downstream target mRNAs of miR-216b-5p by StarBase v2.0 and found that miR-216b-5p could bind to the 3′ UTR region of SKIL (Fig. 5A). Next, dual-luciferase reporter assay showed that the transfection of miR-216b-5p in HCT-116 and SW480 cells could markedly dwindle the luciferase activity with SKIL 3′ UTR WT transfection compared to miR-NC, while the luciferase activity of SKIL 3′ UTR MUT was not significantly affected by over-expression of miR-216b-5p (Fig. 5B, C). To understand the role of SKIL in CRC, we measured SKIL expression in CRC at mRNA and protein levels by qRT-PCR and western blot. The data showed that the mRNA and protein levels of SKIL were upregulated in CRC tissues and cells (Fig. 5D–G). Additionally, overexpression of miR-216b-5p in HCT-116 and SW480 cells inhibited SKIL expression, including mRNA and protein levels (Fig. 5H–K). Given that circ_0029803 could direct target miR-216b-5p, we speculated whether circ_0029803 could regulate the expression of SKIL by targeting miR-216b-5p. The results indicated that when circ_0029803 was silenced in HCT-116 and SW480 cells, the mRNA and protein levels of SKIL were declined, and the effect of si-circ_0029803 on SKIL expression was reversed by anti-miR-216b-5p (Fig. 5L–O).

### SKIL could reverse the biological functions mediated by miR-216b-5p in CRC cells

**Fig. 6:**
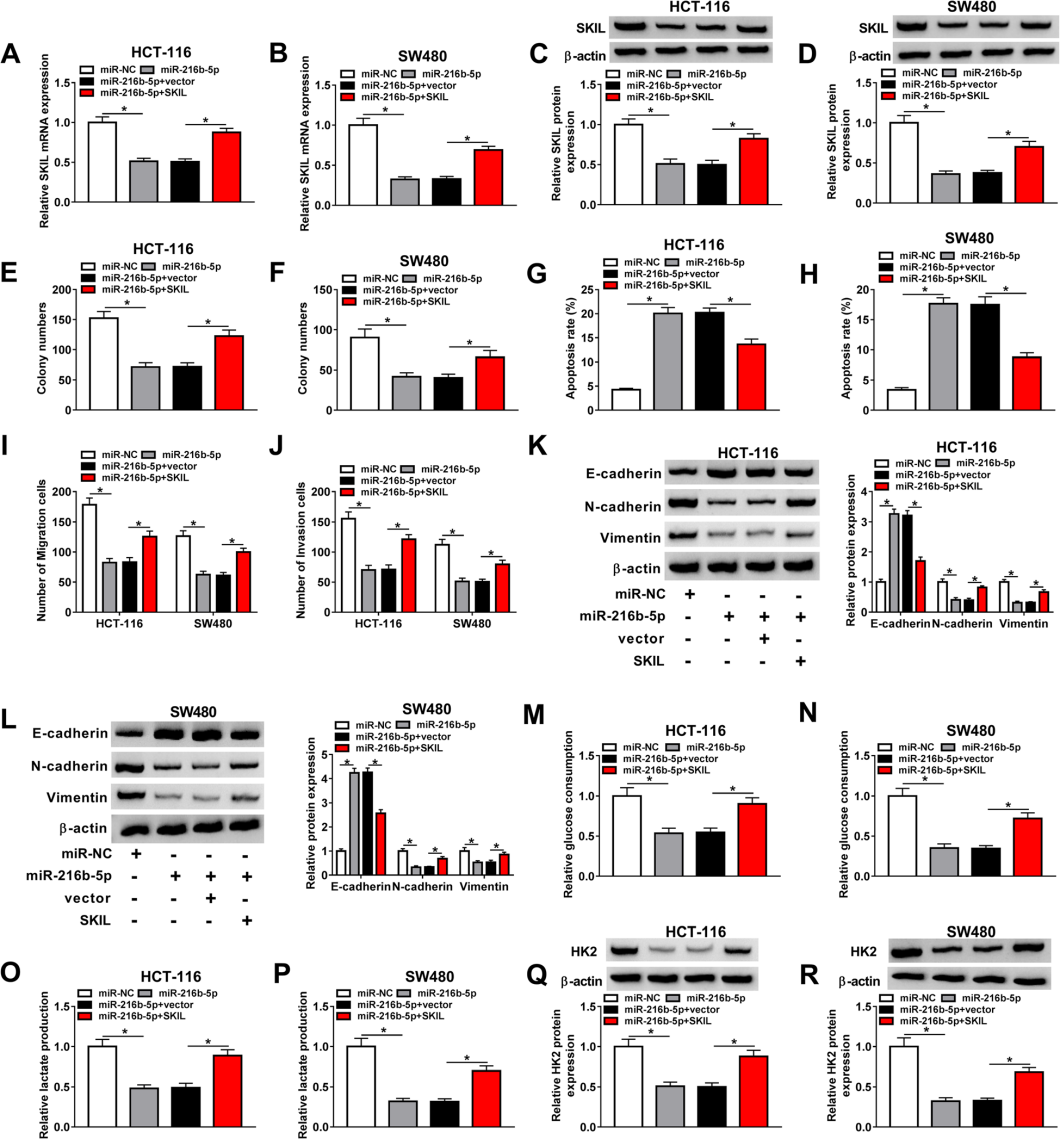
SKIL could reverse the biological functions mediated by miR-216b-5p in CRC cells. **A**–**D** SKIL mRNA and protein levels in HCT-116 and SW480 cells transfected with miR-NC, miR-216b-5p, and miR-216b-5p + vector or miR-216b-5p + SKIL were examined using qRT-PCR and western blot, respectively. **E**–**J** The proliferation, apoptosis rate, migration, and invasion of transfected HCT-116 and SW480 cells were assessed by colony formation, flow cytometry, and Transwell assays, respectively. **K**, **L** The levels of E-cadherin, N-cadherin, and Vimentin in transfected HCT-116 and SW480 cells were measured by western blot. **M**–**P** Glucose consumption and lactate production of transfected HCT-116 and SW480 cells were measured by a glucose and lactate assay kit. **Q**, **R** The protein level of HK in transfected HCT-116 and SW480 cells was also measured by western blot. *P<0.05

To determine whether miR-216b-5p regulated the biological behavior of CRC cells by SKIL, rescue experiments were conducted. HCT-116 and SW480 cells were transfected with the following four groups: miR-NC, miR-216b-5p, miR-216b-5p + vector, and miR-216b-5p + SKIL, and the transfection efficiency was verified by qRT-PCR and western blot (Fig. 6A–D). The results showed that miR-216b-5p overexpression retarded clone formation (Fig. 6E, F), migration, and invasion (Fig. 6I, J), and promoted apoptosis (Fig. 6G, H) of HCT-116 and SW480 cells; however, these effects could be weakened by overexpressing SKIL. Also, upregulation of miR-216b-5p hampered EMT in HCT-116 and SW480 cells, which was reflected in the increase of E-cadherin protein expression and the decrease of N-cadherin and Vimentin protein levels, while the inhibition of miR-216b-5p on EMT was eliminated by overexpression of SKIL (Fig. 6K, L). As with si-circ_0029803, overexpression of miR-216b-5p impeded glucose consumption (Fig. 6M, N), lactate production (Fig. 6O, P), and HK2 protein expression (Fig. 6Q, R) in HCT-116 and SW480 cells, and SKIL could also invert these effects.

### Circ_0029803 knockdown inhibited the growth of CRC tumors by regulating miR-216b-5p/SKIL axis in vivo

**Fig. 7:**
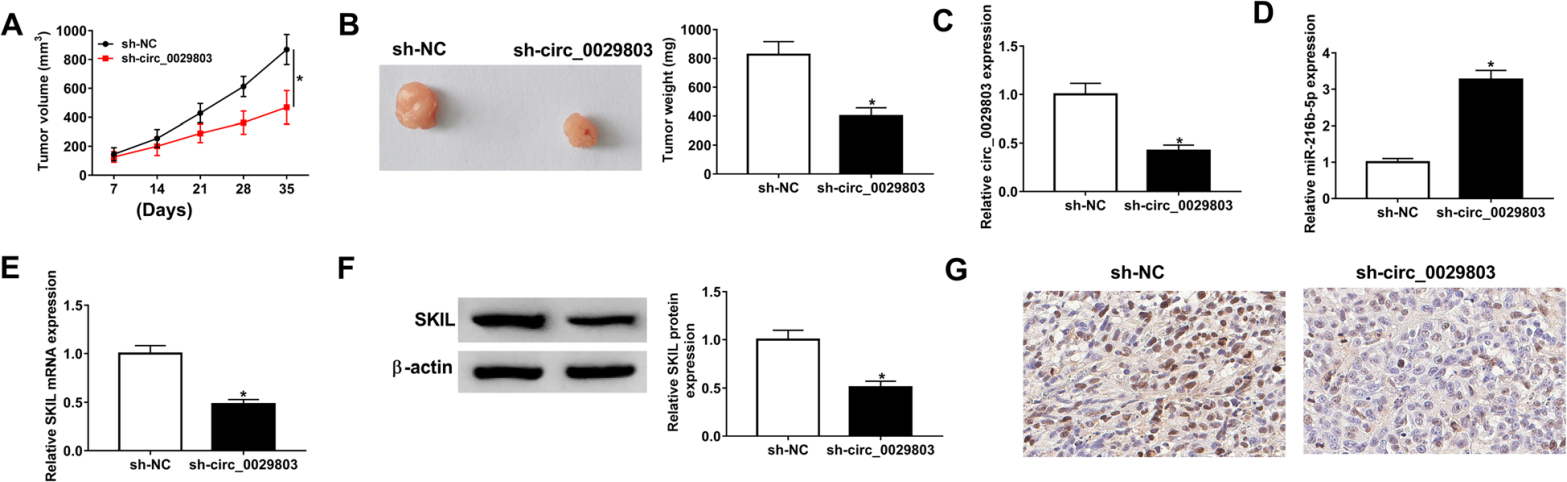
Circ_0029803 knockdown inhibited the growth of CRC tumors by regulating miR-216b-5p/SKIL axis in vivo. After SW480 cells transfected with sh-circ_0029803 or control sh-NC were inoculated into the nude mice. **A**, **B** The tumors’ volume and weight in sh-circ_0029803 group and sh-NC group were measured. **C**–**E** The levels of circ_0029803, miR-216b-5p, and SKIL detected in tumor tissues were examined by qRT-PCR. **F** The protein level of SKIL was also detected in tumor tissues by western blot. **G** The abundance of SKIL in tumor tissues was identified by IHC staining analysis. *P<0.05

To further explore the effect of circ_0029803 on CRC cells in vivo, SW480 cells stably transfected with sh-circ_0029803 or sh-NC were injected subcutaneously into nude mice, and the tumor volumes were measured weekly after injection. As demonstrated in Fig. 7A, B, the volume and weight of tumors in the sh-circ_0029803 group were significantly smaller than those in the negative control. Meanwhile, examined by qRT-PCR, circ_0029803 (Fig. 7C) expression was notably decreased in sh-circ_0029803 group compared with that in the sh-NC group. In contrast, miR-216b-5p showed high expression in the sh-circ_0029803 group (Fig. 7D). SKIL mRNA (Fig. 7E) and SKIL protein (Fig. 7F) were poorly expressed in sh-circ_0029803 group compared with those in the sh-NC group by qRT-PCR and western blot assays. Consistently, the expression of SKIL was also declined in the sh-circ_0029803 group by IHC staining analysis (Fig. 7G). The above data indicated that silencing circ_0029803 hampered tumor growth in vivo by modulating miR-216b-5p/SKIL axis.

## Discussion

As a new ncRNA, circRNA is implicated in various physiological and pathological processes of human cancers [27]. Some circRNAs with abnormal expression have also been found in CRC, such as circ_0026344 [28], circ_0021977 [29], and circ_0004585 [30]. These circRNAs could regulate the growth and metastasis of tumor cells as well as the occurrence of tumors by acting as a sponge for miRNA. Previously, circ_0029803 was found to be overexpressed in CRC tissues through RNA-Seq, but the result has not been verified. In our study, circ_0029803 was confirmed to be increased in CRC tissues and cells by qRT-PCR, and we notarized its existence through RNase R treatment. Additionally, CRC patients exhibiting high circ_0029803 expression had shorter overall survival than those patients exhibiting low circ_0029803 expression, suggesting that circ_0029803 might be a pivotal regulator of CRC.

Subsequently, cell function tests showed that interfering with circ_0029803 retarded proliferation, migration, and invasion of CRC cells, but promoted apoptosis. EMT is characterized by the loss of epithelial features and the acquisition of mesenchymal phenotypes, leading to cancer metastasis and development [31]. E-cadherin, N-cadherin, and Vimentin were considered to be EMT markers [32]. Glycolysis reflects the extent to which cells metabolize, and is characterized by glucose consumption and lactate production. Moreover, HK2 is a key enzyme in glycolysis [33]. When circ_0029803 was knocked down, the levels of N-cadherin and Vimentin were decreased, and E-cadherin expression level was elevated. Besides, glucose consumption, lactate production, and HK2 protein level were reduced in CRC cells, suggesting that EMT and glycolysis were inhibited. Based on these results, we believed that circ_0029803 might be an oncogenic driver in CRC.

CircRNA functions as a sponge of miRNAs, thus releasing mRNA transcripts that are directly targeted by miRNAs, which is widely accepted [34]. There were binding sites between circ_0029803 and miR-216b-5p, and we confirmed that miR-216b-5p was a target of circ_0029803. Then, we measured miR-216b-5p expression in CRC, and miR-216b-5p expression was found to be declined in CRC tissues and cells. The rescue experiments indicated that the suppressive effect caused by si-circ_0029803 on the progression of CRC cells was reversed after miR-216b-5p inhibition. These results suggested that circ_0029803 acted as a sponge of miR-216b-5p to promote the progression of CRC cells in vitro.

Subsequently, we found that SKIL was a target of miR-216b-5p. In CRC, SKIL was upregulated, and its expression was actively regulated by circ_0029803 and negatively modulated by miR-216b-5p, implying that circ_0029803 could modulate SKIL expression by serving as a ceRNA of miR-216b-5p. SKIL has been reported to play a dual role in different cancers [35]; it has both anticancer and carcinogenic activities depending on other genetic changes in the tumor [36]. In our data, overexpression of miR-216b-5p suppressed proliferation, migration, invasion, EMT, and glycolysis of CRC cells and induced apoptosis, while these effects were counteracted by overexpressing SKIL, suggesting that SKIL played a role as an oncogene in CRC. In accordance with previous data, Ye et al. demonstrated that lncRNA SNHG14 facilitated the tumorigenesis and metastasis of CRC by increasing SKIL expression through sponging miR-32-5p [37]. In vivo experiments showed that circ_0029803 knockdown impeded the growth of CRC tumors by reducing SKIL expression and enhancing miR-216b-5p expression.

Our study was the first to preliminarily exploit the role of circ_0029803 in CRC, and we provided a new mechanism to partly clarify how circ_0029083 functioned in CRC. However, there were still some limitations. For example, additional miRNAs targeted by circ_0029083 were not investigated, and the potential oncogenic pathways involved in the circ_0029083-mediated networks were lacking. Future work should focus on these points to further disclose the functional role of circ_0029083 in CRC.

## Conclusion

**Fig. 8:**
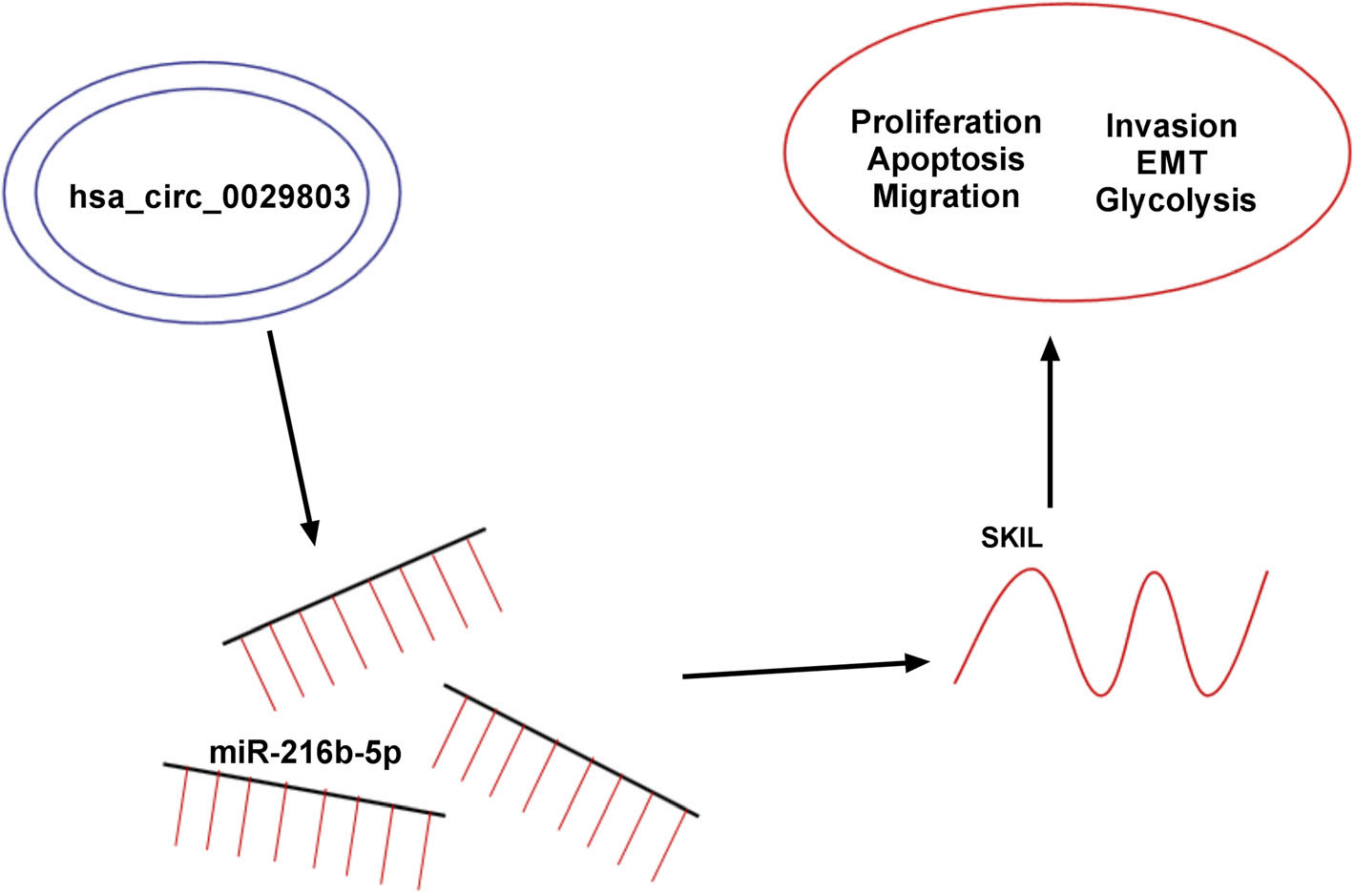
Circ_0029803 promoted the proliferation, migration, invasion, EMT, and glycolysis of CRC cells via targeting miR-216b-5p to regulate SKIL

Our results were the first time to demonstrate that circ_0029803 was augmented in CRC tissues and cells as an oncogene. Mechanically, circ_0029803 modulated SKIL expression through competitive binding with miR-216b-5p. Functionally, circ_0029803 promoted proliferation, migration, invasion, and EMT as well as hindered apoptosis of CRC cells by regulating miR-216b-5p/SKIL axis (Fig. 8). Therefore, circ_0029803 might be a promising molecular marker for the diagnosis of CRC.

## Data Availability

Data sharing is not applicable to this article as no datasets were generated or analyzed during the current study.

## Abbreviations

CRC: Colorectal cancer
SKIL: Ski-oncogene-like
qRT-PCR: Quantitative real-time polymerase chain reaction
circRNAs: Circular RNA
ncRNA: Non-coding RNA
miRNA: MicroRNA
EMT: Epithelial-mesenchymal transformation
MRE: MicroRNA response elements
